# Genetic variation in early fitness traits across European populations of silver birch (*Betula pendula*)

**DOI:** 10.1093/aobpla/plaa019

**Published:** 2020-05-25

**Authors:** Aida Solé-Medina, Katrin Heer, Lars Opgenoorth, Phillip Kaldewey, Darius Danusevicius, Eduardo Notivol, Juan J Robledo-Arnuncio, José A Ramírez-Valiente

**Affiliations:** 1 Department of Forest Ecology & Genetics, INIA-CIFOR, Madrid, Spain; 2 Escuela Internacional de Doctorado, Universidad Rey Juan Carlos, Móstoles, Spain; 3 Conservation Biology, Philipps Universität Marburg, Marburg, Germany; 4 Department of Ecology, Philipps Universität Marburg, Marburg, Germany; 5 Biodiversity and Conservation Biology, Swiss Federal Research Institute WSL, Birmensdorf, Switzerland; 6 Faculty of Forest Science and Ecology, Vytautas Magnus University, Akademija, Kaunas, Lithuania; 7 Unidad de Recursos Forestales, CITA, Avda. Montañana 930, Zaragoza, Spain

**Keywords:** Early mortality, local adaptation, population differentiation, regeneration, seed germination, seed mass, seedling emergence

## Abstract

Given that the ecological niche of tree species is typically narrower for earlier life stages, intraspecific genetic variation at early fitness traits may greatly influence the adaptive response of tree populations to changing environmental conditions. In this study, we evaluated genetic variation in early fitness traits among 12 populations of *Betula pendula* from a wide latitudinal range in Europe (41–55°N). We first conducted a chamber experiment to test for population differences in germination and the effect of pre-chilling treatment on seed dormancy release. We then established three common gardens spread across the species latitudinal range in order to evaluate levels of quantitative genetic variation and genotype-by-environment interaction at different early life traits. Our results showed significant variation in chamber germination rates among populations (0–60 %), with southern populations exhibiting lower germination. Pre-chilling treatments did not generally improve germination success. Population seedling emergence rates in the field were correlated with chamber germination rates, though being an order of magnitude lower, with an average ranging from 0 to 1.3 % across gardens. Highly significant variation was found in field emergence rates among populations, and between seed-crop years within populations, but not among families within populations. Populations differed in seedling height, diameter, slenderness and budburst date, with significant among-family variation. Population latitude was positively associated with chamber germination rate and with seedling emergence rate in one of the central field sites. Overall, genetic, environmental and demographic factors seem to influence the observed high levels of variation in early fitness traits among *B. pendula* populations. Our results suggest limited regeneration capacity for the study species under drier conditions, but further field trials with sufficient replication over environments and seed crops will improve our understanding of its vulnerability to climate change.

## Teaser

Does the origin of the seeds imply differences in early fitness traits? Are the first stages of the regeneration process affected by the environment? Do the origin of the seeds (genotype) and the environment interact? Do the differences in emergence rates and growth traits follow any clinal pattern? Our results on *Betula pendula* common garden experiments across Europe suggest that the answer to all these questions is ‘yes’. Our study also points out the limited regeneration capacity of this species under dry environments. Check out our paper ‘Genetic variation in early fitness traits across European populations of silver birch (*Betula pendula*)’ to learn more!

## Introduction

Climate change models predict drier and warmer summers for Mediterranean regions in the next decades (Kovats *et al.* 2014). In Central Europe, increases in temperature and considerable contrasts in precipitation between dry and wet seasons are also predicted (EEA 2012; Collins *et al.* 2013). These conditions will result in more severe hydrological and soil moisture droughts in many areas, especially in summer months (Wong *et al.* 2011; Samaniego *et al.* 2018). As a consequence, demographic dynamics and species distribution ranges might be considerably altered (Iverson *et al.* 2004; Dyderski *et al.* 2018). In the northern edge of the range, the increase in temperature is expected to broaden the suitable habitat and distribution of many forest tree species (Eggers *et al.* 2008; Nadeau and Urban 2019). On the contrary, populations from the southern edge are expected to suffer from size reductions and increased isolation, making them particularly vulnerable to climatic changes (Jump *et al.* 2006; Peñuelas *et al.* 2007).

There is a need to assess the levels of intraspecific genetic variation in fitness-related traits, and its environmental determinants, in order to evaluate the potential and the drivers of adaptive evolution to climate change (Alberto *et al.* 2013). Adaptive evolution requires the existence of heritable genetic variation at fitness-related traits within and among populations, as it increases both the response to new selective pressures and the frequency of potentially pre-adapted alleles (Jump *et al.* 2009; Kremer *et al.* 2014). Common gardens have long been used to disentangle environmental and genetic effects on phenotypic variability (Savolainen *et al.* 2007; White *et al.* 2007; Alberto *et al.* 2013). However, most common garden studies conducted under natural and semi-natural conditions have focussed on juvenile and adult trees, established from seedlings initially grown in optimal nursery conditions, thus neglecting the impact of natural selective pressures during early life stages (Gibson *et al.* 2016). The early stages of the life cycle, and particularly the transition from seeds to seedlings, are crucial for the regeneration niche and have enormous consequences on population dynamics (Jackson *et al.* 2009; Donohue *et al.* 2010; Walck *et al.* 2011). Predictions on future species distributions based exclusively on adult tree traits could underestimate range contraction risks, given that the ecological niche is typically narrower in early life stages (Jackson *et al.* 2009). Thus, improving our understanding on intraspecific genetic variation in early fitness traits, such as dormancy, germination and seedling establishment rates, is essential to elucidate the potential ability of tree populations to regenerate and persist under climate change.

In this study, we examined the extent to which populations of *Betula pendula*, a widely distributed tree species in Europe, differed in early fitness traits under contrasting environmental conditions. This wind-pollinated and wind-dispersed broadleaved species has a continuous distribution across Central and Northern Europe (Atkinson 1992). It is also found in the Mediterranean Basin, where it is restricted to mountain ranges (Beck *et al.* 2016). Seeds are dispersed in late summer-autumn and stay dormant until spring, when conditions for germination and seedling establishment are more favourable (Vanhatalo *et al.* 1996). Dormancy duration and the rates of germination and seedling survival are critical parameters in the regeneration process (Donohue *et al.* 2010). The main climatic factors limiting the species recruitment are low temperatures in northern Europe (Holm 1994a, b) and summer drought in southern regions, similarly to other European temperate and boreal tree species such as *Betula alba* (Sanz *et al.* 2011) and *Pinus sylvestris* (Castro *et al.* 2004).

We expect that different selective pressures across the species range have resulted in adaptive genetic divergence across *B. pendula* populations (Savolainen *et al.* 2007). We hypothesize clinal, rather than abrupt, geographic genetic divergence among populations, given the continue distribution and high levels of gene flow in this species (Atkinson 1992; Alberto *et al.* 2013). We expect differences in early fitness traits among seed-crop years related with the great inter-annual variation in reproduction investment in this species (Rousi *et al.* 2011; Gallego Zamorano *et al.* 2018). Differences among populations in dormancy, germination, growth and phenology traits associated with latitudinal and altitudinal clines have already been found in northern populations (e.g. Myking and Heide 1995; Viherä-Aarnio and Velling 2008; Midmore *et al.* 2015), but there is little information on range-wide genetic variation at early fitness traits assayed under natural conditions, especially for southern and mid-low latitude populations.

Our specific objectives were: (i) to explore whether *B. pendula* exhibits among-population genetic variation at early life traits along a latitudinal gradient, (ii) to explore the potential geographic and environmental factors associated with the observed population genetic divergence, (iii) to determine the extent to which the growing environment alters patterns of population variation at early life traits (i.e. genotype-by-environment interaction) and (iv) to examine levels of within-population genetic variation at those traits.

## Materials and Methods

### Plant material

**Figure 1.**
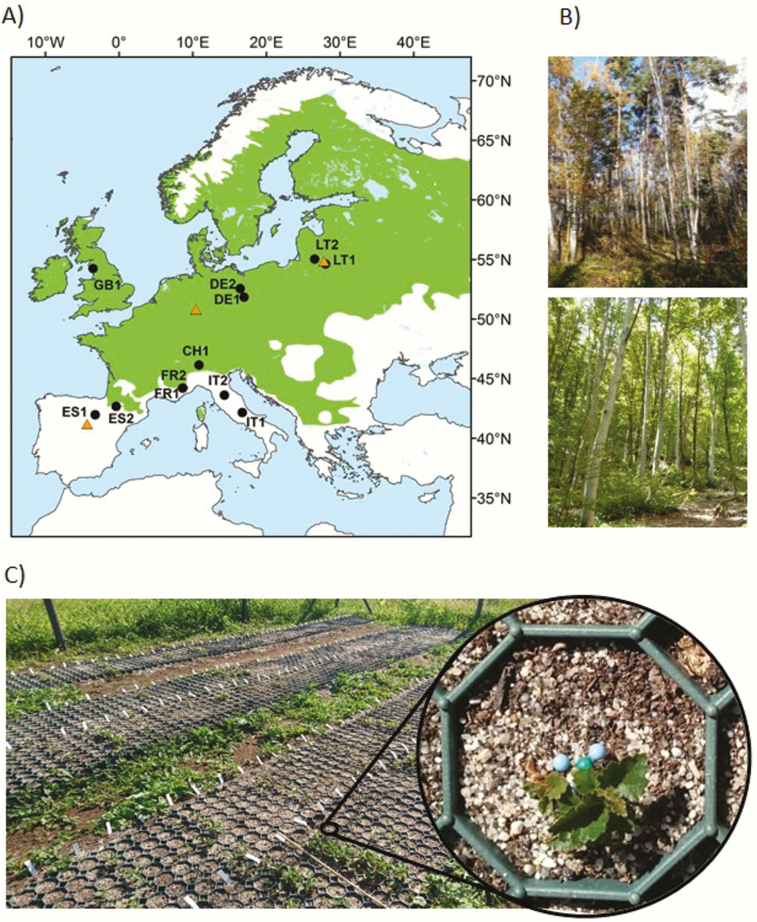
(A) Location of sampled *Betula pendula* populations with available seeds (black circles) and the sites of the common garden trials (orange triangles). The green area indicates the species distribution range (EUFORGEN 2009). (B) Natural *B. pendula* populations DE1 (top) and IT1 (bottom). (C) German garden and zoom-in of a cell with seedlings.

Twelve natural populations of *B. pendula* were selected for the study throughout the species distribution range in Europe (Fig. 1; Table 1). Seeds were collected in the summers of 2016 and 2017 from 11–25 open-pollinated and randomly selected trees within each population, separated at least 30 m apart. For populations ES2 and DE1, seed lots were obtained for both 2016 and 2017 crops, which were used to explore variation in early fitness traits among seed-crop years within populations (Table 1). Unless specified otherwise, ES2 and DE1 refer to the seed crop from 2016. Seeds were stored at 4 °C in a dry environment until sowing.

**Table 1.** Population code, country, latitude, longitude, altitude, annual mean temperature (AMT) and annual precipitacion (AP) of the studied *Betula pendula* populations. Next columns represent the number of experimental units used for the analyses of germination rate in chamber (GR), emergence rate (ER) in Spain and Germany and survival, as well as the number of seedlings for the analysis of growth and phenology traits. See Materials and Methods for more details. Climatic data for the period 1979–2013 obtained from CHELSA (Karger *et al.* 2017). ^a^Populations with two seed crops available (2016 and 2017) for the analysis of temporal variation. ^b^Populations with maternal family structure for the analysis of intrapopulation genetic variation. ^c^Populations assayed only in control treatment in the chamber experiment.

Population	Country	Latitude	Longitude	Altitude (m)	AMT (°C)	AP (mm)	GR	ER	Survival	Growth	Phenology
ES1	Spain	41°58′N	2°37′W	1271	8.8	531	12	18	5		
ES2	Spain	42°40′N	0°19′W	988	9.1	1001	12^a^	120^a,b^	26^a,b^	29	29
IT1	Italy	42°09′N	13°37′E	1498	6.9	963	12	18	7		
IT2	Italy	43°36′N	11°42′E	1084	8.9	1134	12	18	8		
FR1	France	44°11′N	7°04′E	1128	6.8	799	12	18	2		
FR2	France	44°12′N	7°05′E	1519	5.1	920	12	18	9	20	18
CH1	Switzerland	46°08′N	8°59′E	1089	8	1815	12	18	2		
DE1	Germany	51°50′N	14°26′E	72	9.6	573	12^a^	90^a,b^	33^b^	127^b^	135^b^
DE2	Germany	52°32′N	14°3′E	55	9.3	532	12				
GB1	UK	54°13′N	3°01′W	31	9.8	1105	3^c^	18	8		
LT1	Lithuania	54°37′N	24°13′E	118	7	661	12	18	9	53	60
LT2	Lithuania	55°01′N	23°0′E	56	7.4	669	12	120^b^	52^b^	212^b^	231^b^

### Chamber experiment

In winter 2018, we conducted a chamber experiment to (i) obtain benchmark germination rates, (ii) assess the effect of moist-chilling on seed dormancy, germination rate and time and (iii) examine whether populations responded differently to its application. We established three treatments with different chilling duration: 15 days (T1), 30 days (T2) and 45 days (T3) and a control treatment, where seeds were not chilled (C). Chilling consisted of adjusting seeds to a moisture level of 33 % at 4 °C in darkness, conditions that have been reported to break dormancy while avoiding seed germination and deterioration (De Atrip and O’Reilly 2007). Seed moisturizing was achieved by adding distilled water on seeds placed in 1.5ml microtubes (Jones and Gosling 1994). After each chilling treatment, 150 seeds per population (6–9 seeds per mother tree) were placed in three different Petri dishes (i.e. 50 seeds per population per block) with moistened filter paper inside. Population GB1 was only essayed in the C treatment due to limited seed availability. Petri dishes were placed in the chamber following a randomized block design. Temperature was set at 15 ± 2 °C, to simulate approximate soil temperatures in the field, and photoperiod to 24 h light. Petri dishes were moistened periodically with distilled water. Germination was monitored three times per week until the end of the experiment (45 days after sowing).

We calculated germination percentage per Petri dish, and mean germination time in days (MGT) as:

$$\begin{array}{l} MGT=\frac{\overset{k}{\underset{i=1}{\sum }}n_{i}t_{i}}{\overset{k}{\underset{i=1}{\sum }}n_{i}} \end{array}$$

where *n*_*i*_ is the number of seeds germinated at the *i*th observation date; *t*_*i*_, the number of days since the beginning of the experiment to the *i*th date; and *k* the total number of dates of observation (Ranal and Santana 2006).

### Common garden trials

#### Experimental design

In spring 2018, three common garden experiments were established across the latitudinal range of *B. pendula* in Europe in Cerezo de Arriba, Spain (41°11′49.8″N, 3°31′17.4″W), Marburg, Germany (50°48′03.6″N, 8°48′24.8″E) and Šlienava, Lithuania (54°51′18.6″N, 24°03′02.9″E) (Fig. 1). The Spanish site was located in the species southern limit, at 1319 m.a.s.l. (metres above sea level), characterized by a temperate oceanic submediterranean climate (Rivas-Martínez *et al.* 2004) (Table 2). The other two sites were located in the centre of the species distribution range, at 325 m.a.s.l. in Germany and 70 m.a.s.l. in Lithuania, representing temperate oceanic climate and temperate continental climate, respectively (Rivas-Martínez *et al.* 2004) (Table 2).

**Table 2.** Monthly precipitation (Pp, in mm) and monthly mean, maximum and minimum temperatures (*T*_mean_, *T*_max_, *T*_min_, in °C) during 3 months after sowing at the three common garden sites. Values correspond to averages for the reference period 1970–2000 obtained from WorldClim (Hijmans *et al.* 2005), and to records for the study year (2018). NA: no available data. ^a^Data available from 15 May.

		May		June		July	
		Reference	2018	Reference	2018	Reference	2018
Lithuania	Pp	49	19	68	58	79	138
	*T*_mean_	12.4	16.9	15.7	17.3	17.1	20.7
	*T*_max_	18.0	23.1	21.3	23.4	19.4	25.4
	*T*_min_	6.8	10.8	10.1	11.3	14.9	13.8
Germany	Pp	67	36.2^a^	69	19.6	67	243
	*T*_mean_	12.4	17.5^a^	15.4	17.9	17.2	21.3
	*T*_max_	17.1	23.8^a^	20.1	23.8	19.7	28.6
	*T*_min_	7.7	12.2^a^	10.6	12.8	14.7	14.2
Spain	Pp	68	NA	45	65	21	2
	*T*_mean_	10.3	NA	15.1	16.0	18.9	18.8
	*T*_max_	16.4	NA	21.2	19.9	23.3	23.9
	*T*_min_	4.2	NA	9.0	12.0	14.6	13.7

In each garden, we established 255 experimental units distributed in three blocks (85 experimental units per block). Each experimental unit consisted of 16 cells of 30 cm^2^ each, delimited with plastic ground-grids that were open in the bottom and lateral walls (Guttagarden®). Grids were filled with a mixture of 50 % fine sand and 50 % peat to homogenize superficial soil conditions across gardens. Seeds from population DE2 were not included in the design because they did not germinate in the chamber (see Results and Table 1). The final design comprised 11 different populations, plus the second-year (2017) seed lots of populations DE1 and ES2. The family structure (i.e. the maternal identity) was maintained in the 2016 seed lots from ES2 (Spain), DE1 (Germany) and LT2 (Lithuania) (Table 1). These three populations were selected based on their proximity (geographically and climatically) to the corresponding common garden sites. Seeds from different mother trees were pooled within each of the remaining seed lots before sowing. Fifty-five out of the 85 experimental units per block corresponded to maternal families of the populations with family structure (1 experimental unit per family per block). The remaining 30 units in each block corresponded to populations without family structure (3 experimental units per population per block). Experimental units were randomized following a 51 × 5 latinized row-column design, using CycDesigN software (Whitaker *et al.* 2002).

#### Seed mass, preparation and sowing

We measured average seed mass per population and per maternal family in the populations without and with family structure, respectively. We used between 0.04 and 0.06 g of seeds per population or maternal family for this estimation (ISTA 2007). Since chilling treatments had a negative impact on both germination rate and time (see Results), seeds used for the common gardens were not chilled. *Betula pendula* typically exhibits high proportions of unviable (empty) seeds, with substantial variation among populations (e.g. 30–70 % in Midmore *et al.* 2015). Reliably separating full and empty *Betula* seeds is a very time-consuming process, unfeasible for the large amounts used in this study. So, aiming to obtain a sufficient and balanced number of emerged seedlings across populations in the field while controlling for the effect on emergence rates of potential variation in empty seed proportions, we adjusted the number of seeds sown per population or maternal family in the common gardens. Based on benchmark chamber germination rates (influenced by the presumably variable proportion of empty seeds in the lots), we estimated the number of seeds per cell needed to expect at least one germinated seed with 95 % probability for each population in each cell, and multiplied this number by a safety factor of 25, considering that field emergence rates might be lower than baseline chamber rates. For maternal families from ES2, DE1 and LT2, we used the averaged population germination rate for these estimates. Seeds were sown in spring 2018 and were watered three times per week for 1 month.

#### Field measurements and fitness estimates

Emergence and survival were monitored three times per week the first month after sowing, every 1–2 weeks the second month and once per month until November 2018. Each day of measurement, we counted the number of seedlings per cell and considered emergence and mortality when the number of seedlings increased or decreased since the last day of measurement, respectively.

#### Growth and phenology traits

At the end of the first growing season (November 2018), height and diameter of the surviving seedlings were measured. We also calculated the slenderness index, a metric of mechanical stability, as height/diameter. At the beginning of the following growing season (mid-May 2019), bud burst was scored every 1–2 days in one seedling per cell when leaf tips were clearly above bud scales (Finn *et al.* 2007). Growth and phenology were only measured in the German garden, because of null emergence and null survival in the Lithuanian and Spanish sites, respectively. Consequently, garden-by-population or garden-by-family interactions were only tested for emergence rates but not for growth or for phenology traits (see also Statistical analyses).

### Statistical analyses

#### Chamber experiment

Binomial mixed effects models with logit link function and lineal mixed effect models were used to test for differences in germination rate (GR) and in mean germination time (MGT) among populations and chilling treatments. Population, treatment and their interaction were included as fixed-effect factors and block as a random-effect factor. Analogous models were implemented to test for differences in GR and MGT between seed-crop years for ES2 and DE1, with seed-crop year and treatment as fixed-effect factors.

#### Common gardens

Both the seedling emergence and survival rates in the common garden sites were averaged per experimental unit (grid of 16 cells) for the analyses. Survival was measured relative to the number of emerged seedlings. Emergence was low overall and both fitness components presented an overdispersed zero-inflated distribution, with many empty cells and most seedlings emerging (and surviving) in small clumps. We, therefore, used generalized additive models (GAMLSS) with a zero-inflated beta distribution (BEINF0) for the analyses of both the emergence and the survival rate. This distribution is a mixture of a discrete value 0 with probability *p*_0_ and a beta distribution *f*_*w*_ ~ Beta(*µ*, *σ*) on the unit interval (0, 1) with probability (1 − *p*_0_). The probability density function of the mixture is thus given by Equation 1:

$$\begin{array}{l} f_{y}(y|\mu ,\sigma ,\nu )=\left\{ \begin{array}{ll} p_{0} & if\;y=0\\ (1-p_{0})f_{w}(y\;|\;\mu ,\sigma )\; & if\;0<y<1 \end{array}\right. \end{array}$$

In the case of the emergence rate, the mean *µ* of the beta distribution is the estimated mean emergence rate *given that* emergence is not zero (which in our experimental setting can be interpreted as the mean emergence rate across cells showing *some* emergence), *σ* is the scale parameter of the beta distribution and *ν* yields the probability *p*_0_ that the emergence rate is zero as *p*_0_ = *ν*/(1 + *ν*) (which in turn can be interpreted here as the estimated proportion of experimental cells not showing any emergence at all). Analogously for the survival rate, *μ* is the estimated proportion of surviving seedlings *given that* survival is not zero (which can be regarded here as the mean survival rate across cells with *some* survival), *σ* is the scale parameter and *ν* yields the probability of zero survival as *p*_0_ = *ν*/(1 + *ν*) (i.e. the estimated proportion of cells where all emerged seedlings died). We tested for differences among populations, gardens and their interaction in emergence and survival rates by modelling *μ* and *ν* parameters. We only modelled the intercept for *σ* parameter, as otherwise convergence was not reached. Models included population, garden and population-by-garden as fixed-effect factors, and block and column both nested within garden as random-effect factors. The resulting models were compared using likelihood-ratio tests. To test for differences in emergence and survival between seed-crop years of ES2 and DE1, we performed one GAMLSS for each of the two populations including seed-crop year, common garden and their interaction as fixed-effect factors, and the same random structure as before. Generalized additive models for emergence rate of the three populations with family structure were analysed separately, with population, garden and population-by-garden as fixed-effect factors, and block nested within garden and family nested within population as random-effect factors. Linear models were used to test the associations between observed emergence rates, estimated *μ* and *p*_0_ in the common gardens and observed chamber germination rates. Linear models were also used to examine associations between observed emergence and survival rates. Mean germination time was not analysed in field conditions since the number of emerged seedlings was very low, which affects the interpretation of this parameter (Ranal and Santana 2006).

Linear mixed models were used for the analyses of growth and phenology traits in the five populations with >25 seedlings alive. Population was included as a fixed-effect factor, and block, column and row as random-effect factors. To avoid the effect of competition, only cells with less than five seedlings were used for the analyses of the growth traits. Comparisons of population means were assessed using Tukey *post hoc* tests. Finally, we also used linear mixed models to analyse growth and phenology traits in populations with family structure. All terms were included as random-effect factors to study their effects on the variance.

The association between trait population means and geographic and environmental variables of the seed provenances was tested using multiple regression. *Betula pendula* is only present in high altitudes in the southern areas and in mid-low altitudes in northern populations. Consequently, latitude and altitude were strongly negatively correlated (*r* = −0.93, *P* < 0.001). Longitude and latitude were also marginally correlated (*r* = 0.55, *P* = 0.054). To avoid multicollinearity, we only included latitude in the multiple regression, as it better represents the geographic structure of the sampled populations. We also included two climatic variables in the multiple regression: mean annual temperature and annual precipitation for the period 1979–2013 obtained from CHELSA (Karger *et al.* 2017). We used linear regressions to test the associations between population trait means and seed mass. A step forward-backward selection was performed. Association among traits was tested using Pearson correlations.

All analyses were conducted using R 2.3.5 (R Core Team 2014). We used the packages ‘lme4’ (Bates *et al*. 2015) for mixed models, ‘emmeans’ (Lenth *et al.* 2019) for *post hoc* tests, ‘Hmisc’ (Harrell 2019) for correlation analyses, ‘gamlss’ (Rigby and Stasinopoulos 2005) for the analyses of traits with zero-inflated distributions (germination and survival), and ‘ggplot2’ (Wickham 2016) and ‘plotly’ (Plotly Technologies Inc. 2005) for graphic representation of the results.

## Results

### Seed germination in the chamber experiment

**Figure 2.**
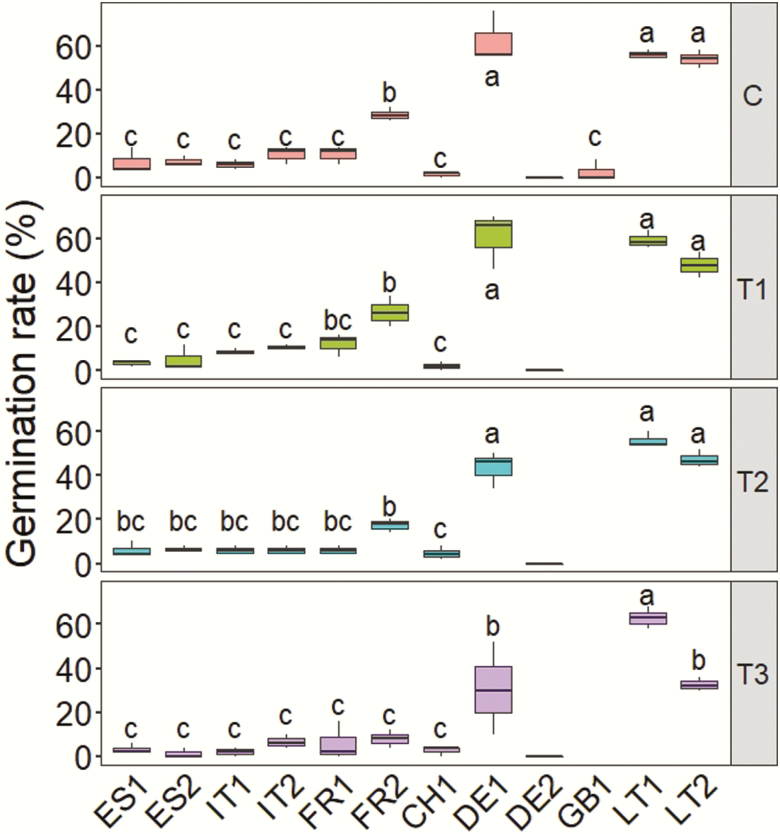
Germination rates for 14 *Betula pendula* populations in controlled chamber conditions under four treatments: a control without pre-chilling (C) and pre-chilling treatments with a duration of 15 days (T1), 30 days (T2) and 45 days (T3). Population GB1 was only tested in the control treatment (C) due to low seed availability. Means that do not share letters indicate significant differences among populations within treatments (*P* < 0.05, Tukey test). Boxes show the median (inside line), the interquartile range (hinges are the 25th and 75th percentiles) and 1.5 times the interquartile range (whiskers). Populations are ordered by latitude (codes as in Table 1).

Mixed models revealed significant differences among chilling treatments in both germination rate (GR) and mean germination time (MGT) **[see****Supporting Information—Table S1****]**. On average, C and T1 treatments resulted in higher GR (mean 17.4 ± standard error 1.50 % and 16.6 ± 1.45 %, respectively) than T2 (12.9 ± 1.21 %) and T3 (8.6 ± 0.9 %). In addition, C and T1 seeds had shorter MGT (13.3 ± 1.23 and 13.7 ± 1.27 days, respectively) compared to T2 and T3 pre-chilled seeds (16.7 ± 1.54 and 14.7 ± 1.39 days, respectively) (data not shown). There were also significant population differences in both traits, and population-by-treatment interaction in GR **[see****Supporting Information—Table S1****]**. Populations LT1, LT2 and DE1 had the highest GR in all treatments, whereas most of the other populations exhibited very low values (Fig. 2). Population DE2 did not germinate in any treatment (Fig. 2). Significant differences in GR among 2016 and 2017 seed crops were found for population DE1 (1.1 % in 2017 versus 49.1 % in 2016) but not for ES2 **[see****Supporting Information—Table S2****]**. Population LT1 had the shortest MGT (10.3 ± 1.1 days), whereas ES1 and CH1 had the longest MGT (19.9 ± 2.02 and 20.4 ± 2.18 days, respectively) (data not shown). GR and MGT were not correlated (*P* = 0.201).

### Seedling emergence in the field

**Figure 3.**
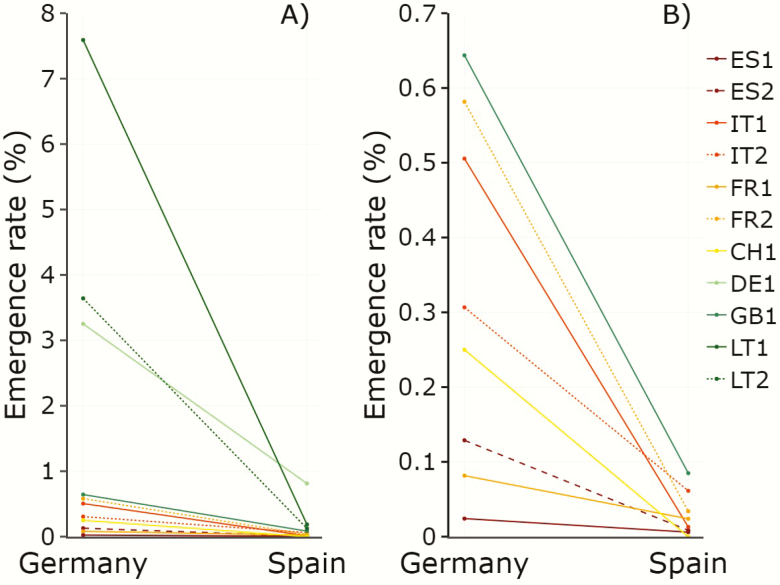
Population means (dots) and reaction norms (lines) for seedling emergence rates of the studied populations of *Betula pendula* in two common gardens in Germany and Spain. Panel (A) shows all studied populations in both common gardens. Panel (B) displays the zoom-in of populations with emergence rates below 1 %. Population codes as in Table 1. Standard errors are not shown for clarity.

No seedling emergence was observed in the Lithuanian site, whereas a total of 383 and 4554 seedlings emerged in the Spanish and German gardens, respectively, which represent 0.1 % and 1.3 % of the estimated seeds sown in each of the two sites. In the two common gardens with emergence, three populations (LT1, LT2 and DE1) showed emergence rates that were an order of magnitude higher than those of the rest (Fig. 3).

Generalized additive models showed significant differences among gardens for emergence rate in both *μ* and *ν* parameters **[see****Supporting Information—Table S3****]**. There were also significant differences among populations in both *μ* and *ν* for seedling emergence, as well as significant population-by-garden interaction in *ν*, but not in *μ***[see****Supporting Information—Table S3****]**. The *μ* parameter, which represents the estimated seedling emergence rate across experimental cells with *some* emergence, ranged between 0.82 % (for ES1) and 7.15 % (for LT1) in the German garden and between 0.20 % (for ES1) and 0.95 % (for DE1) in the Spanish garden **[see****Supporting Information—Table S4****]**. In the German garden, the estimated probability of zero emergence, *p*_0_ (calculated using *ν* in Equation 1) ranged between 77.8 % for FR1 and CH1 and 0.0 % for FR2 and LT1, indicating that all cells in the experiment for the two latter populations had some emergence **[see****Supporting Information—Table S4****]**. In the Spanish garden, *p*_0_ ranged between 100 % (i.e. all cells had zero emergence) for CH1, and 33 % for IT2, FR1, GB1 and LT1, indicating that these four populations exhibited some emergence in two-thirds of the experimental cells **[see****Supporting Information—Table S4****]**. There were correlations between observed population seed germination rates in the chamber and observed population seedling emergence rates, correlations that were significant at the German garden (*r* = 0.91, *P* < 0.001) and marginally significant in the Spanish garden (*r* = 0.63, *P* = 0.050). There was no correlation between observed population emergence rates in the two common gardens (*r* = 0.43, *P* = 0.213).

Generalized additive models revealed significant differences in emergence rates between the two different seed-crop years of the DE1 population in the German garden, both in *μ* and *ν***[see****Supporting Information—Table S5****]**, ranging between 3.25 % and 0.01 % for the 2016 and 2017 seed crops, respectively. In the Spanish garden, the null emergence of the seed crop from 2017 of DE1 contrasted with the relatively high emergence rate found for seed crop 2016 (0.95 %). No significant differences in emergence were found between the seed-crop years of ES2 in any of the two gardens **[see****Supporting Information—Table S5****]**. Generalized additive models performed on populations with family structure for emergence rates did not reveal significant family effects for *ν* or *μ* (Table 3), whereas differences among populations were significant for both parameters (Table 3).

**Table 3.** Results of the GAMLSS for *Betula pendula* populations with family structure (ES2, DE1, LT2) for seedling emergence rate in the Spanish and German gardens and for seedling survival rate in the German garden. *μ*_1_ is the estimated proportion of emerged seedlings in cells with some emergence and *ν*_1_ yields the probability of no emergence (*p*_0_), as *p*_0_ = *ν*/(1 + *ν*) (i.e. the estimated proportion of cells without any emergence); *μ*_2_ is the estimated proportion of surviving seedlings in cells where survival is not zero and *ν*_2_ yields the probability of no survival (*p*_0_), as *p*_0_ = *ν*/(1 + *ν*) (i.e. the estimated proportion of cells where all emerged seedlings died). LRT: likelihood-ratio test, df: degrees of freedom. Significant values are in bold type (*P* < 0.05).

Trait	Parameter	Factor	df	LRT	*P*
Emergence rate	*μ*_1_	Residual	313		
		Garden	**1**	**11.02**	**<0.001**
		Block (garden)	1	0.72	0.396
		Population	**2**	**40.52**	**<0.001**
		Family (population)	1	2.22	0.136
		Pop × garden	2	2.68	0.261
	*ν*_1_	Garden	1	2.68	0.102
		Block (garden)	**1**	**4.53**	**0.033**
		Population	**2**	**18.33**	**<0.001**
		Family (population)	1	1.22	0.268
		Pop × garden	**2**	**10.95**	**0.004**
Seedling survival	*μ*_2_	Residual	100		
		Block	1	3.15	0.076
		Population	2	5.19	0.075
		Family (population)	1	2.44	0.119
	*ν*_2_	Block	1	0.72	0.397
		Population	**2**	**14.59**	**<0.001**
		Family (population)	1	1.93	0.164

### Seedling survival in the field

**Figure 4.**
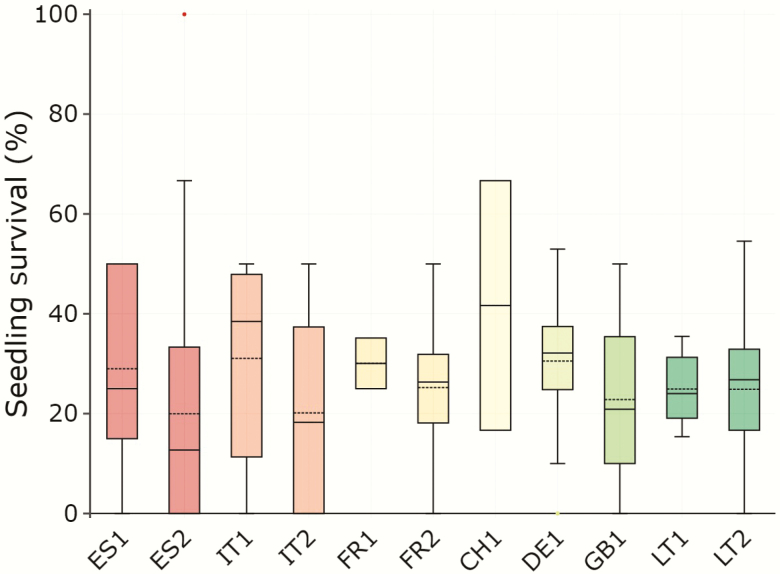
Observed seedling survival rate after the first growing season for *Betula pendula* populations in a common garden experiment in Germany. Generalized additive models showed differences among populations in *ν* (*P* = 0.010) but not in *μ* (*P* = 0.171), indicating that populations differed in the estimated proportion of cells without survival (*p*_0_), but not in the estimated proportion of cells with at least one seedling alive at the end of the experiment **[see****Supporting Information—Table S6****]**. Boxes show the median (inside solid line), the mean (inside dashed line), the interquartile range (hinges are the 25th and 75th percentiles), 1.5 times the interquartile range (whiskers) and outliers (dots). Population codes as in Table 1. Populations are ordered by latitude, colours as in Fig. 3.

All seedlings that emerged in the Spanish garden died before the end of the experiment. By contrast, 1274 seedlings (29 % of emerged seedlings) survived in the German garden. Observed seedling survival rates varied from 20 % to 42 % for ES2 and CH1, respectively (Fig. 4). Differences among populations were significant for the *ν* parameter but not for *μ***[see****Supporting Information—Table S6****]**, indicating significant population variation in the estimated proportion of cells without survival (*p*_0_, calculated from *ν*), though not in the estimated survival rate for cells exhibiting seedlings alive at the end of the experiment. Observed seedling emergence and seedling survival probability were not correlated (*r* = −0.12, *P* = 0.723), i.e. seeds that tended to germinate more did not tend to produce more viable seedlings. The proportion of sown seeds resulting in alive seedlings at the end of the experiment was mostly determined by the emergence rate (*r* = 0.99, *P* < 0.001).

The highest and lowest survival rates corresponded to both seed crops from ES2 (2016: 20 % and 2017: 43.7 %), although GAMLSS did not show significant differences among them in *μ* or *ν***[see****Supporting Information—Table S5****]**. Seedlings from the 2017 seed crop of DE1 did not survive in the German garden, precluding testing for differences between seed crops for this population.

Seedling survival rate GAMLSS performed on populations with family structure did not show a significant family effect in *μ* or *ν*, whereas a highly significant population effect was observed for *ν* (Table 3).

### Field growth and phenology traits

**Figure 5.**
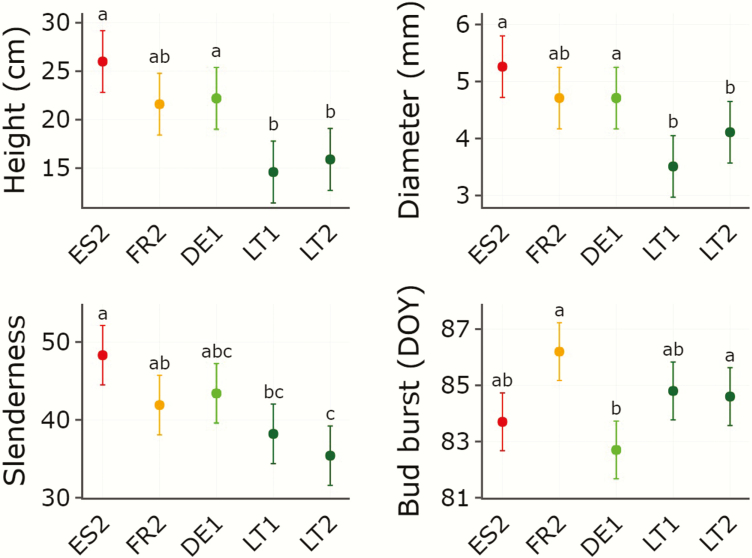
Means and standard errors for height, diameter, slenderness and bud burst measured in seedlings of *Betula pendula* populations growing in the German common garden experiment. Only populations with >25 seedlings still alive in spring 2019 were used. Populations are ordered by latitude. Values that do not share letters are significantly different (*P* < 0.05, Tukey test). Population codes as in Table 1. Population colours as in Figs 3 and 4.

There were only five populations with >25 seedlings alive to conduct mixed models for growth and phenology traits (see Table 1). Results showed significant differences among populations in height, diameter, slenderness and the day of bud burst (**see****Supporting Information—Table S7**; Fig. 5). Height was strongly correlated with diameter (*r* = 0.97, *P* = 0.005) and slenderness (*r* = 0.94, *P* = 0.015). No significant correlation was found between diameter and slenderness (*r* = 0.85, *P* = 0.067). Correlations between bud burst and growth traits were not significant.

**Figure 6.**
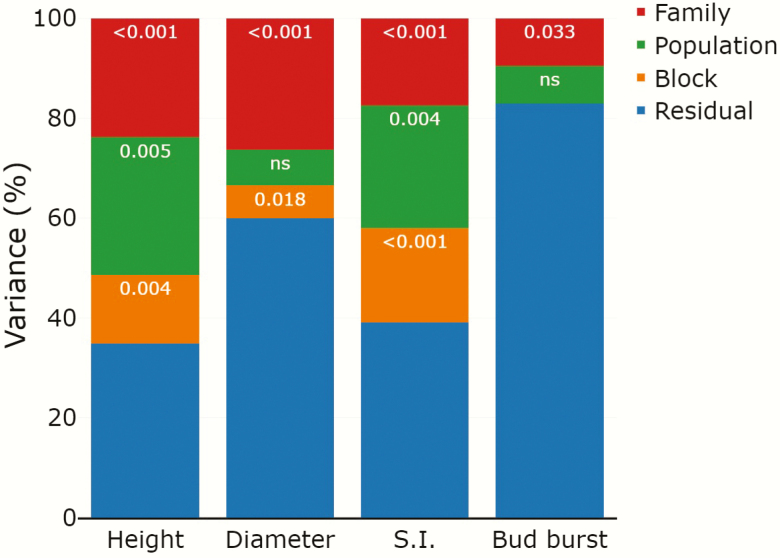
Percentage of phenotypic variance of early growth and phenology traits (height, diameter, slenderness index: SI and bud burst) explained by mixed model factors block, population and maternal family within populations in *Betula pendula* common gardens. The numbers inside the bars are *P*-values of the effects, ns indicates a non-significant effect. There was no block variance for bud burst.

Analyses for growth and phenology traits at the family level were only conducted for populations LT2 and DE1, as population ES2 had a very low number of seedlings (none of the families had >6 seedlings alive and more than half of the families had between 0–2 seedlings). Mixed models for height and slenderness showed significant family and population effects that explained together >40 % of the variance (Fig. 6). Mixed models for diameter showed a significant family effect that explained 26.3 % of the variance, while the population effect was not significant (Fig. 6). For the date of bud burst, differences among families were significant, although this effect explained <10 % of the variance. No significant differences among populations were found for this trait (Fig. 6).

### Climatic and geographic patterns

**Figure 7.**
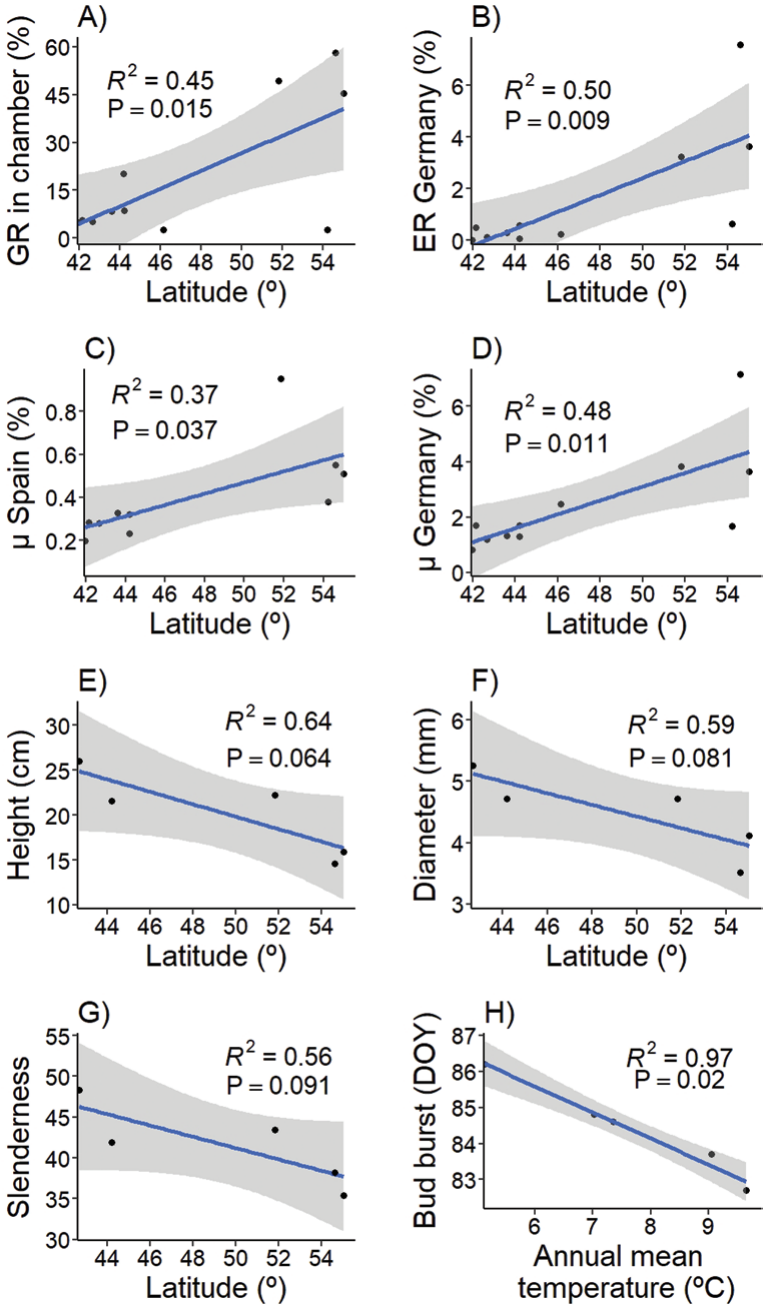
Linear regressions between the latitude of *Betula pendula* populations and (A) population chamber germination rate, GR; (B) seedling emergence rate, ER, in the German site; (C) the estimated proportion of emerged seedlings in cells with *some* emergence (*μ*) in the Spanish site; (D) *μ* in the German site; (E) seedling height; (F) seedling diameter; and (G) seedling slenderness. Panel (H) shows the linear regression between annual mean temperature of the populations and bud burst date. Dots represent population means. Grey shaded areas indicate 95 % confidence intervals. Emergence rate in Spain was not significantly associated with any geographic or climatic variable (not shown). Growth and phenology traits (height, diameter, slenderness and bud burst) were only tested in the German garden. Standard errors of population trait means are not shown for clarity.

Multiple regression analyses showed a significant positive association between latitude and chamber germination rate, and between latitude and field seedling emergence rate at the German garden (Fig. 7A and B). A significant positive association between latitude and *μ* (i.e. the mean seedling emergence rate across cells with *some* emergence) was found in both the Spanish and German common gardens (Fig. 7C and D). No significant associations with climatic or geographic variables were found for seed mass, emergence rate in Spain, probability of no germination (*p*_0_) in any of the gardens or seedling survival in Germany (which was only measured there). Associations between growth traits and climate variables were not significant, although marginally significant negative relationships were found between latitude and height, diameter and slenderness (Fig. 7E–G). No association was found between date to bud burst measured in the common garden and latitude. In contrast, the date to bud burst was negatively correlated with the annual mean temperature at the population of origin (Fig. 7H). Linear regressions also showed significant positive associations between seed mass and germination rates in chamber **[see****Supporting Information—Fig. S1a****]** and between seed mass and emergence rates in both the Spanish and German gardens **[see****Supporting Information—Fig. S1b****]**, but not between seed mass and growth or phenology traits (data not shown).

## Discussion

The present paper investigated genetic variation at early fitness traits among and within *B. pendula* populations spread over a large portion of the distribution range of the species, including southern margins. By performing chamber experiments under controlled conditions and common gardens trials at three semi-natural sites across a climatic gradient, we detected strong population variation in germination and emergence rates as well as in growth and phenology traits throughout the first year of the life cycle.

### Germination rate in the chamber experiment

Germination is one of the most drastic transitions, and earliest fitness components, in plant life cycles (Donohue *et al.* 2010). In our chamber experiment, germination rates were generally low, but with substantial differences among populations (Fig. 2). Three populations exhibited germination rates above 50 %, whereas 9 out of 12 populations had germination rates below 10 % in all pre-chilling treatments (Fig. 2). Differences in germination rates among populations of *B. pendula* have been previously reported, even at smaller geographical scales (Holm 1994a). Similar to our results, Midmore *et al.* (2015) predicted markedly high differences in germination rates among populations, ranging between 0 and 100 % among populations from 47°N to 60°N. Similar patterns of population-level variation have been found in other *Betula* species (e.g. Bevington 1986; Reyes *et al.* 1997). Differences in germination rates among populations could have resulted from different proportions of filled seeds, which is supported by the strong positive correlation between seed mass and germination rates **[see****Supporting Information—Fig. S1****]**. Midmore *et al.* (2015) showed that, on average, southern populations had a higher percentage of empty seeds (~77 %) than northern populations (~28 %). Southern *B. pendula* populations are generally more isolated, have smaller size, lower density and lower levels of genetic variation, which could have limited ovule fertilization rates because of more irregular pollen availability and/or inbreeding depression (Holm 1994a; Palmé *et al.* 2003). In addition, resource scarcity and warm and dry conditions during seed maturation could have resulted in smaller, less vigorous embryos in southern populations. In all, lower germination rates in southern populations appear to be greatly determined by demographic and environmental factors.

Other factors could have been also important in the observed germination patterns. Specifically, *B. pendula* is a masting species with wide annual variation in the quantity and quality of seed crops (Atkinson 1992). In our study, differences in germination between seed crops from 2016 versus 2017 were strikingly high for population DE1, suggesting that investment in reproduction and environmental factors could influence the proportion of viable seeds. Finally, it is worth noting that dormancy is a typical characteristic of birch seeds (Myking and Heide 1995). Differences in germination among populations could have also resulted from differences in dormancy duration or different environmental requirements to break their dormancy. For instance, Midmore *et al.* (2015) showed that populations of *B. pendula* from higher latitudes were more sensitive to pre-chilling compared to populations from lower latitudes, particularly under colder temperatures. In our study, in contrast, germination rates were largely unaffected by pre-chilling. These results, together with the similar patterns of emergence rates observed in field conditions (see next section), suggest that potential differences in dormancy among populations are unlikely to be major determinants of observed variation in population germination rates.

### Emergence and survival rates in the common gardens

Three populations located in the centre of the species distribution area had emergence rates an order of magnitude higher than those of the rest (Fig. 3). Demographic, environmental and genetic factors are expected to have shaped population patterns of emergence rates in the field, as discussed for germination rates. Field emergence, however, was strikingly lower than chamber germination in all populations. This is consistent with Tylkowski (2012), who found that *B. pendula* seedling emergence rates from seeds sown in containers were lower than the corresponding germination rates in chamber. We expected the Lithuanian site to be the most suitable for *B. pendula* according to climate niche models (Beck *et al.* 2016). However, null seedling emergence was found in Lithuania. Since seeds of this species require a continuously moisturized seedbed for germination (Gordon 1992), the exceptionally warm and dry 2018 spring in Lithuania probably minimized germination and precluded any seedling emergence in this site, despite the initial watering. These results suggest that extreme climatic events can hamper natural regeneration even in the central core of the distribution of *B. pendula*, and that seedling establishment is a phase very sensitive to climatic conditions (Rousi *et al.* 2011). The very low emergence rate and the subsequent total seedling mortality observed for all provenances at the Spanish site might also have resulted from the dry conditions during spring and summer. This site is located in the southern edge of the species, in an area characterized by a mountainous Mediterranean climate where summer storms play an important role in long-term regeneration of temperate species. Rainfall in spring of 2018 was higher than average in the area, but rains were irregular, with short dry and hot intermediate periods during which soil moisture was considerably reduced. Moreover, the summer was much drier than average (Table 2). These results suggest that drought strongly limits seedling emergence and survival of *B. pendula*, and therefore that drier conditions expected in the southern edge of the species range in the next decades will hinder natural regeneration in this area (Humphries *et al.* 1982; Hynynen *et al.* 2010; Beck *et al.* 2016; Pliūra *et al.* 2019).

Climatic conditions in the German site seemed more favourable, even if seedlings were exposed to low precipitation that originated some soil water deficit in mid-summer. Emergence rates in Germany did not reach the seed germination rates observed in the chamber, but still were significantly higher than those observed in Spain and Lithuania. The German garden was in addition the only of the three gardens where some seedlings survived until the end of the experiment. The comparatively high survival rate of emerged seedlings (28 %) relative to the low emergence rate (1.3 %) suggests that selection pressures at early life stages in *B. pendula* are particularly strong during the seed-to-seedling transition. Furthermore, the lack of significant population differentiation in seedling survival rates among populations within experimental sites suggests, in accordance with van Andel (1998), that the effect of environmental conditions on early seedling mortality substantially exceeds the one of the genetic background. These results highlight the importance of including the germination (and not only the seedling/sapling) stage in field trials conducted to assess adaptive genetic variation and to identify the life stages that represent the major bottlenecks for recruitment (Walck *et al.* 2011).

### Growth and phenology traits

Our results showed population differences in growth and phenology traits. Growth in height and diameter tended to be higher in populations from lower latitudes (Fig. 7E–G). Previous studies have also reported negative latitudinal clines in growth traits in *B. pendula* at smaller spatial scales (Velling 1979; Myking and Heide 1995; Viherä-Aarnio and Velling 2008). This latitudinal pattern could reflect local adaptation to differences in the length of the growing period. Viherä-Aarnio *et al.* (2005) found that, under a common environment, southern populations of the species were able to keep growing in days with shorter photoperiods, therefore prolonging their growing season in autumn. Phenology in spring could also play a role in the differences in growing season length across populations. In fact, our results showed that populations of *B. pendula* differed in the timing of bud burst. However, this trait was associated with annual temperature but not with latitude. Furthermore, Li *et al.* (2002) found 40-day differences among southern and northern populations of *B. pendula* in the date of growth cessation, while differences in growth initiation in spring were of only 4–7 days. These observations suggest a minor contribution of early phenology to growth variation among populations of *B. pendula*.

Different photosynthetic capacity might also have contributed to observed growth differences among populations. Previous studies have reported gas exchange and growth potential variation across genotypes of *B. pendula* (Possen *et al.* 2011). Aspelmeier and Leuschner (2004) observed that *B. pendula* genotypes from areas with drier summers had higher gas exchange rates, including higher stomatal conductance and photosynthesis, in both well-watered and dry conditions. These authors suggested that this strategy would favour fast growth and competitiveness in drought-prone habitats.

Contrary to expectations based on previous studies, seed mass was not significantly associated with growth traits. Seed mass has been generally observed to play an important role in early growth even at intraspecific level (e.g. Oleksyn *et al.* 1998; Rose *et al.* 2009; Ramírez-Valiente and Robledo-Arnuncio 2015). One possible reason for the absence of association between seed mass and growth could be low statistical power, resulting from the small number of populations that had seedlings alive at the end of the experiment. Other reason is that seed mass was determined mostly by empty seed proportions, and not by viable seed mass differences. Finally, it could be that traits such as growth phenology and gas exchange capacity would be more important than seed mass for growth potential in this species.

### Within-population variation at early fitness traits

Most forest trees show high within-population genetic variation (Hamrick 2004), particularly widely distributed species (e.g. Ramírez-Valiente *et al.* 2014, 2015). Studies in *B. pendula* have shown significant intrapopulation genetic variation in growth and phenology traits in adult and juvenile trees (Baliuckienë and Baliuckas 2006; Rousi and Heinonen 2007; Baliuckas and Pliūra 2008). We also found differences among families within populations in early growth traits and in the timing of bud burst, with the family effect explaining between 9 and 25 % of the total phenotypic variance for these traits. The variance explained by the family effect was indeed larger than that of the population effect for diameter and bud burst. These results align with those of previous studies suggesting that intrapopulation genetic variance in *B. pendula* growth and phenology traits is an important component of the intraspecific genetic diversity in this species. However, the substantial levels of genetic variance in bud burst reported within *B. pendula* populations might be insufficient to enable fast enough adaptation to the expected rate of change in temperature (Billington and Pelham 1991; Possen *et al.* 2014). On the other hand, we failed to detect significant variation in the rates of seedling emergence and survival among families within populations (Fig. 4). One possible reason for this negative result is that the low germination rates resulted in low statistical power for the analysis of intrapopulation genetic variation. Another possible explanation is that the environmental conditions might have imposed a very strong selective pressure (reflected by the low emergence rates), thus reducing the expression of intrapopulation genetic variability.

In conclusion, our study revealed extremely low germination and seedling survival of *B. pendula*, particularly under dry conditions, suggesting limited future regeneration capacity if drought conditions increase, especially in the southern margins. Populations greatly differed in early fitness components, with significant intrapopulation variation detected for growth and phenology traits but not for emergence and survival rates. Genetic differences and other non-genetic factors, such as demography, climate and maternal effects, could have shaped the observed population variation patterns. These results, along with high among-year variation in seed production and germination, highlight the difficulty of achieving general conclusions concerning tree early fitness traits. Further studies under sufficiently replicated environmental conditions will be necessary to test for plastic responses, ideally using seeds collected in different years, to improve our understanding on the potential regeneration niche and vulnerability of forest trees species such as *B. pendula* to climate change.

## Data

Phenotypic data used in this study are available at https://zenodo.org/record/3865210

## Supporting Information

Table S1. Results of the mixed models for seed germination rate and mean germination time in the chamber experiment.

Table S2. Results of the mixed models for seed germination rate in the chamber experiment of seed-crop years 2016 and 2017.

Table S3. Results of the generalized additive models (GAMLSS) for emergence rate.

Table S4. Estimated values for emergence rate parameters using generalized additive models (GAMLSS).

Table S5. Results of the generalized additive models (GAMLSS) for emergence and survival rate for 2016 and 2017 seed crops of populations ES2 and DE1.

Table S6. Results of the generalized additive models (GAMLSS) for survival rate.

Table S7. Results of linear mixed models for height, diameter, slenderness and bud burst.

Figure S1. Regression models for the relationship between seed mass and chamber germination and field emergence rates.

plaa019_suppl_Supplementary_Material

plaa019_suppl_Supplementary_Information

## Sources of Funding

This project has received funding from the European Union’s Horizon 2020 research and innovation programme under grant agreement no. 676876 (GenTree project). A.S.-M. was supported by a PhD grant from the Subdirección General de Investigación y Tecnología of the Instituto Nacional de Investigación y Tecnología Agraria y Alimentaria (FPI-SGIT2016-01).

## Conflict of Interest

None declared.

## Contributions by the Authors

J.J.R.-A. conceived the idea; J.J.R.-A., A.S.-M., J.A.R.-V. and E.N. designed the study; A.S.-M., K.H., L.O., P.K., D.D., J.A.R.-V and J.J.R.-A. established the experiment and collected data; A.S.-M. conducted the data analysis and wrote the manuscript; all authors read, commented and approved the manuscript.
