# Supplementary material for: Genetic variation in early fitness traits across European populations of silver birch (Betula pendula)
Source: AoB Plants. 2020 May 25;12(3):plaa019. doi: 10.1093/aobpla/plaa019 (PMC7320878; doi:10.1093/aobpla/plaa019)

Supporting information

Table S1. Results of the mixed models for seed germination rate (GR) and mean germination time (MGT) in the chamber experiment. Population (Pop), chilling treatment (Treat) and its interaction were fixed-effect factors and block was random-effect factor. A binomial distribution an logit link function were used for seed germination rate. Df: degrees of freedom.

	Factor	Df	χ^2	*P*
GR	Residual	78		
	Block	**1**	**5.87**	**0.015**
	Treatment	**3**	**65.18**	**< 0.001**
	Population	**9**	**1445.41**	**< 0.001**
	Pop × Treat	**27**	**50.20**	**0.004**
MGT	Residual	71		
	Block	**1**	**11.26**	**< 0.001**
	Treatment	**3**	**10.73**	**0.013**
	Population	**9**	**68.94**	**< 0.001**
	Pop × Treat	27	20.94	0.789

Table S2. Results of the mixed models for seed germination rate (GR) in the chamber experiment of seed-crop years 2016 and 2017 of populations ES2 and DE1. Seed-crop year (Year), chilling treatment (Treat) and its interaction were fixed-effect factors and block was random-effect factor. A binomial distribution an logit link funciton was used for seed germination rate. Df: degrees of freedom.

	ES2			DE1		
Factor	Df	χ^2	P	Df	χ^2	P
Residual	16			16		
Block	1	0.00	0.999	1	1.30	0.254
Year	1	0.07	0.797	**1**	**450.12**	**< 0.001**
Treatment	**3**	**8.32**	**0.040**	**3**	**39.68**	**< 0.001**
Year × Treat	3	2.44	0.487	3	6.31	0.098

Table S3. Results of the generalized additive models (GAMLSS) for emergence rate in the German and Spanish gardens. Garden, population and the interaction between population and garden (Pop × Garden) were included as fixed-effect factors, while block and column (both nested within garden) were included as random-effect factors. μ is the estimated proportion of emerged seedlings in cells with some emergence and ν yields the probability of no emergence (p_0), as $p_0=\nu/(1+\nu)$ (i.e. the estimated proportion of cells without any emergence). LRT: likelihood ratio test, Df: degrees of freedom. Significant values are in bold type ($P < 0.05$).

	Factor	Df	LRT	*P*
	Residual	418		
	Garden	**1**	**21.74**	**< 0.001**
	Block (Garden)	1	0.87	0.351
μ	Column (Garden)	1	1.76	0.185
	Population	**10**	**85.26**	**< 0.001**
	Pop × Garden	9	14.02	0.122
	Garden	**1**	**47.11**	**< 0.001**
	Block (Garden)	**1**	**4.36**	**0.037**
ν	Column (Garden)	**1**	**4.40**	**0.036**
	Population	**10**	**83.33**	**< 0.001**
	Pop × Garden	**9**	**35.72**	**< 0.001**

Table S4. Estimated parameter values (± standard error) for emergence rate μ (in percentage), ν and p_0 in *B. pendula* populations established in the German and Spanish common gardens, using generalized additive models (GAMLSS). μ is the estimated proportion of emerged seedlings in cells with some emergence, and p_0 is the estimated probability of no emergence calculated as p_0=100*ν/(1+ ν) (i.e. the estimated proportion of cells without any emergence).

Population	German site			Spanish site		
	μ	v	p_0	μ	v	p_0
ES1	0.82 ± 0.36	0.80 ± 0.54	44.44	0.20 ± 0.11	2.00 ± 1.41	66.7
ES2	1.19 ± 0.22	1.31 ± 0.34	56.67	0.28 ± 0.11	9.00 ± 3.87	90.0
IT1	1.69 ± 0.58	0.29 ± 0.23	22.22	0.28 ± 0.19	3.50 ± 2.81	77.8
IT2	1.34 ± 0.44	0.13 ± 0.13	11.11	0.33 ± 0.12	0.50 ± 0.35	33.3
FR1	1.31 ± 0.86	3.50 ± 2.81	77.78	0.23 ± 0.09	0.50 ± 0.35	33.3
FR2	1.70 ± 0.51	0.00	0.00	0.32 ± 0.17	2.00 ± 1.41	66.7
CH1	2.47 ± 1.47	3.50 ± 2.81	77.78	-	-	100
DE1	3.84 ± 0.50	0.36 ± 0.12	26.67	0.95 ± 0.12	0.55 ± 0.17	35.6
GB1	1.66 ± 0.53	0.13 ± 0.13	11.11	0.38 ± 0.14	0.50 ± 0.35	33.3
LT1	7.15 ± 1.44	0.00	0.00	0.55 ± 0.19	0.50 ± 0.35	33.3
LT2	3.65 ± 0.39	0.15 ± 0.06	13.33	0.51 ± 0.11	2.75 ± 0.80	73.3

Table S5. Results of the Generalized Additive Models (GAMLSS) for emergence rate in the German and Spanish gardens and seedling survival rate in the German garden for seed crops of 2016 and 2017 of populations ES2 and DE1. Population was included as fixed-effect factors and block, and column both nested within garden were included as random-effect factors. μ is the estimated proportion of emerged seedlings in cells with *some* emergence and ν yields the probability of no emergence (p_0), as $p_0 = \nu/(1+\nu)$ (i.e. the estimated proportion of cells without any emergence). LRT: likelihood ratio test, Df: degrees of freedom. Significant values are in bold type ($P < 0.05$).

			ER Spain			ER Germany			Survival Germany		
		Factor	Df	LRT	*P*	Df	LRT	*P*	Df	LRT	*P*
ES2	μ	Residual	60			60			21		
		Block	1	2.13	0.145	1	1.59	0.208	1	2.77	0.096
		Column	1	0.52	0.469	1	0.21	0.643	**1**	**4.13**	**0.042**
		Year	1	0.29	0.590	1	1.42	0.233	1	2.37	0.124
	ν	Block	1	1.23	0.267	**1**	**5.55**	**0.019**	1	0.18	0.672
		Column	1	0.34	0.561	1	2.88	0.090	**1**	**5.35**	**0.021**
		Year	1	0.01	0.921	1	0.01	0.938	1	0.65	0.420
DE1	μ	Residual	No emergence seed crop 2017			45			No survival seed crop 2017		
		Block				1	1.51	0.219			
		Column				1	0.12	0.732			
		Year				**1**	**11.82**	**<0.001**			
	ν	Block				1	1.12	0.291			
		Column				**1**	**6.22**	**0.013**			
		Year				**1**	**6.01**	**0.014**			

Table S6. Results of the Generalized Additive Models (GAMLSS) for survival (relative to the number of seedlings emerged) in the German common garden. Block and Column were included as random-effect factors. Population was included as a fixed-effect factor. μ is the estimated proportion of surviving seedlings in cells where survival is not zero and ν yields the probability of no survival (p_0), as $p_0=\nu/(1+\nu)$ (i.e. the estimated proportion of cells where all emerged seedlings died). LRT: likelihood ratio test, Df: degrees of freedom. Significant values are in bold type ($P < 0.05$).

	Factor	Df	LRT	*P*
	Residual	134		
	Block	**1**	**4.84**	**0.028**
μ	Column	**1**	**7.03**	**0.008**
	Population	10	14.04	0.171
	Block	1	0.13	0.719
ν	Column	**1**	**4.96**	**0.026**
	Population	**10**	**23.31**	**0.010**

Table S7. Results of the linear mixed models for height, diameter, slenderness and bud burst in seedlings of *B. pendula* populations growing in the German garden. Population was included as fixed-effect factor. Block, Row and Column were included as random effect factors. LRT: likelihood ratio test, Df: degrees of freedom. Significant values are in bold type ($P < 0.05$).

	Factor	LRT	Df	*P*
Height	Population	**11.0**	**4**	**< 0.001**
	Block	0.8	1	0.3816
	Row	**103.0**	**1**	**< 0.001**
	Column	**28.7**	**1**	**< 0.001**
Diameter	Population	**5.9**	**4**	**< 0.001**
	Block	0.3	1	0.578
	Row	**91.4**	**1**	**< 0.001**
	Column	**43.1**	**1**	**< 0.001**
Slenderness	Population	**10.8**	**4**	**< 0.001**
	Block	3.6	1	0.056
	Row	**121.3**	**1**	**< 0.001**
	Column	0.5	1	0.496
Bud burst	Population	**5.0**	**4**	**< 0.001**
	Block	0.0	1	0.999
	Row	**8.6**	**1**	**0.003**
	Column	**11.9**	**1**	**< 0.001**

Figure S1. Regression models for the relationship between seed mass and germination rate in chamber (a) and observed seedling emergence rate in the German (green line) and Spanish common gardens (red line) (b). Grey shaded area indicates 95 % confidence interval.

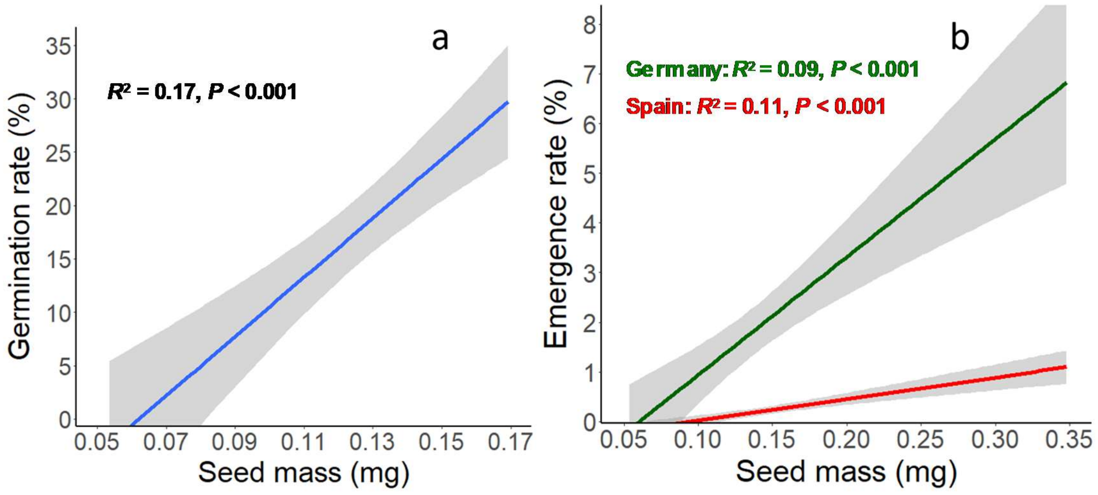

Supplement: plaa019_suppl_Supplementary_Information [file plaa019_suppl_supplementary_information.pdf]
